# A case report of sepsis and death caused by *Parvimonas micra*, a rare anaerobe

**DOI:** 10.3389/fpubh.2022.994279

**Published:** 2022-09-20

**Authors:** Yuhang Jiang, Weichao Qin, Jian Li, Yuan Gao, Yan Zeng

**Affiliations:** ^1^Department of Laboratory Medicine, Chongqing University Jiangjin Hospital, Chongqing, China; ^2^Department of Laboratory Medicine, Central Hospital of Jiangjin District, Chongqing, China; ^3^Department of Laboratory Medicine, Jiangjin District Maternity and Child Health Hospital, Chongqing, China

**Keywords:** *Parvimonas micra*, bloodstream infection, blood culture, septicemia, matrix-assisted laser desorption/ionization time-of-flight mass spectrometry, molecular diagnostic technique, next-generation sequencing, diabetes mellitus

## Abstract

*Parvimonas micra* is a type of Gram-positive anaerobic cocci widely distributed in the oral cavity, gastrointestinal tract, respiratory tract, and female reproductive system mucosa. It is a conditional pathogen that can cause infections in the human oral cavity, wounds, and other areas as well as sepsis. In this case report, the patient's immune system was compromised by various underlying diseases and a pulmonary infection, which led to the entry of *P. micra* infection into the bloodstream. *P. micra* is a slow-growing organism (When a bloodstream infection occurs, flagging an anaerobic bottle of blood culture as positive will usually take >48 h), which makes it hard to secure timely blood culture results. Our patient's poor physical condition eventually led to sepsis, and she died after 5 days in the hospital.

## Introduction

*Parvimonas micra* (hereafter *P. micra*) are Gram-positive cocci recognized as a type of oral pathogen (1). In recent years, due to the use of matrix-assisted laser desorption/ionization time-of-flight mass spectrometry and molecular diagnostic techniques, reports of infections with *P. micra* are increasing, but deaths caused by *P. micra* remain rare and, as far as we know, there are no reports in the literature containing laboratory identification details and morphological characteristics of *P. micra*. In the present paper, therefore, we offer more details about the laboratory identification process (including molecular biology techniques) and morphological characteristics of this organism. The described case was confirmed to be the first report of death in China due to sepsis and shock caused by a *P. micra* bloodstream infection that resulted in multiorgan failure of the patient. We hope this case report will encourage more clinicians and laboratory medicine staff to be aware of the potential threat represented by this bacterium.

## Case report

The patient was a 53-year-old woman with rheumatoid arthritis for >20 years who was on long-term pain medication (the exact type of medication was not known) and who had a history of hypertension for >1 year (her systolic blood pressure was normally maintained at around 150 mmHg). She had high blood glucose results tested in other hospitals, but she did not undergo a confirmatory test for diabetes before she was admitted to our hospital. The patient had previously presented with lower back pain and weakness in both lower limbs with no apparent cause 1 month prior to admission to our hospital and she had been treated in another hospital for a period of time (she failed to provide the specific type of medication used for treatment). Within a few days, she had new symptoms of coughing with phlegm and black stools, and her low back pain had not improved significantly. Thus, she came to our hospital for further treatment. After additional inquiry about the patient's condition, the patient stated that she had not had any recent trauma, and had not urinated in the past 3 days. Then the patient was subsequently taken to the intensive care unit for emergency treatment. The patient finally died of multi-organ failure due to infectious shock even after 5 days of anti-infective treatment in the intensive care unit. This study was approved by the ethics committee at the Chongqing University Jiangjin Hospital, and the patient's family member provided written consent for the publication of the report.

## Clinical findings

The patient was admitted with an acute painful appearance, and physical examination revealed wet rales in both lungs and significant percussion pain in the low back. Physical examination of the patient at admission revealed a body temperature of 36.2°C, pulse 136 beats/min, respiration 20 breaths/min, and blood pressure 114/70 mmHg. An emergency chest computed tomography scan revealed scattered irregular patchy and streaky shadows in both lungs and bilateral pleural effusions, fractures of the right scapula, sternum, bilateral multiple ribs and sacrum.

## Laboratory examinations and clinical diagnosis

The patient's arterial blood gas analysis revealed the following values: pH, 7.41; PaCO_2_, 27 mmHg; PaO_2_, 54 mmHg; whole blood lactic acid, 3.5 mmol/L; and fasting blood glucose, 11.2 mmol/L. A complete blood count revealed the following: hemoglobin, 104 g/L; platelet count, 225 × 10^9^/L; and neutrophil % count, 87.10%. The patient's biochemical parameters are shown in Table 1, and her white blood cell (WBC) count, procalcitonin concentration, and CRP level at various times during her hospital stay are shown in Table 2. Corona virus disease 2019 (COVID-19) nucleic acid test was negative. The above-mentioned tests combined with the patient's chest CT findings suggested a preliminary diagnosis of pulmonary infection, type 1 respiratory failure, and type 2 diabetes mellitus, as there was no history of trauma, the patient was considered to have a pathological fracture due to infection. The results of multiple sputum cultures were negative; a total of two sets of blood cultures were taken from the upper and lower extremities of the patient at the time of fever and sent for culturing. The anaerobic bottles were all positive, and the culture results were all *P. micra* (however, the patient had already unfortunately passed away by the time the bacterial identification results were obtained).

**Table 1:** Biochemical parameters of this patient.

**Biochemical parameters items**	**Results**	**Biochemical parameters items**	**Results**
Blood urea nitrogen	14.4 mmol/L	interleukin-6	410.40 pg/mL
Blood uric acid	668 μmol/L	Cystatin-C	1.35 mg/L
Glycosylated hemoglobin	7.8%	Beta-2-microglobulin	7.33 mg/L
Hypersensitivity C-reactive (CRP) protein	294.05 mg/L	Prealbumin	13.7 mg/L

**Table 2:** Trends in inflammatory indicators during the patient's hospitalization.

	**Day 1**	**Day 2**	**Day 3 (Antibiotic changed)**	**Day 4**	**Day 5**
WBC count (×109/L)	21.19	14.77	14.17	17.94	18.36
CRP (mg/L)	>200	>200	>200	>200	>200
PCT (ng/mL)	2.93	2.55		2.55	1.63

WBC, white blood cell; CRP, C-reactive protein; PCT, procalcitonin.

A blank spot indicates that the item was not tested at that day.

### Bacterial identification

**Figure 1:**
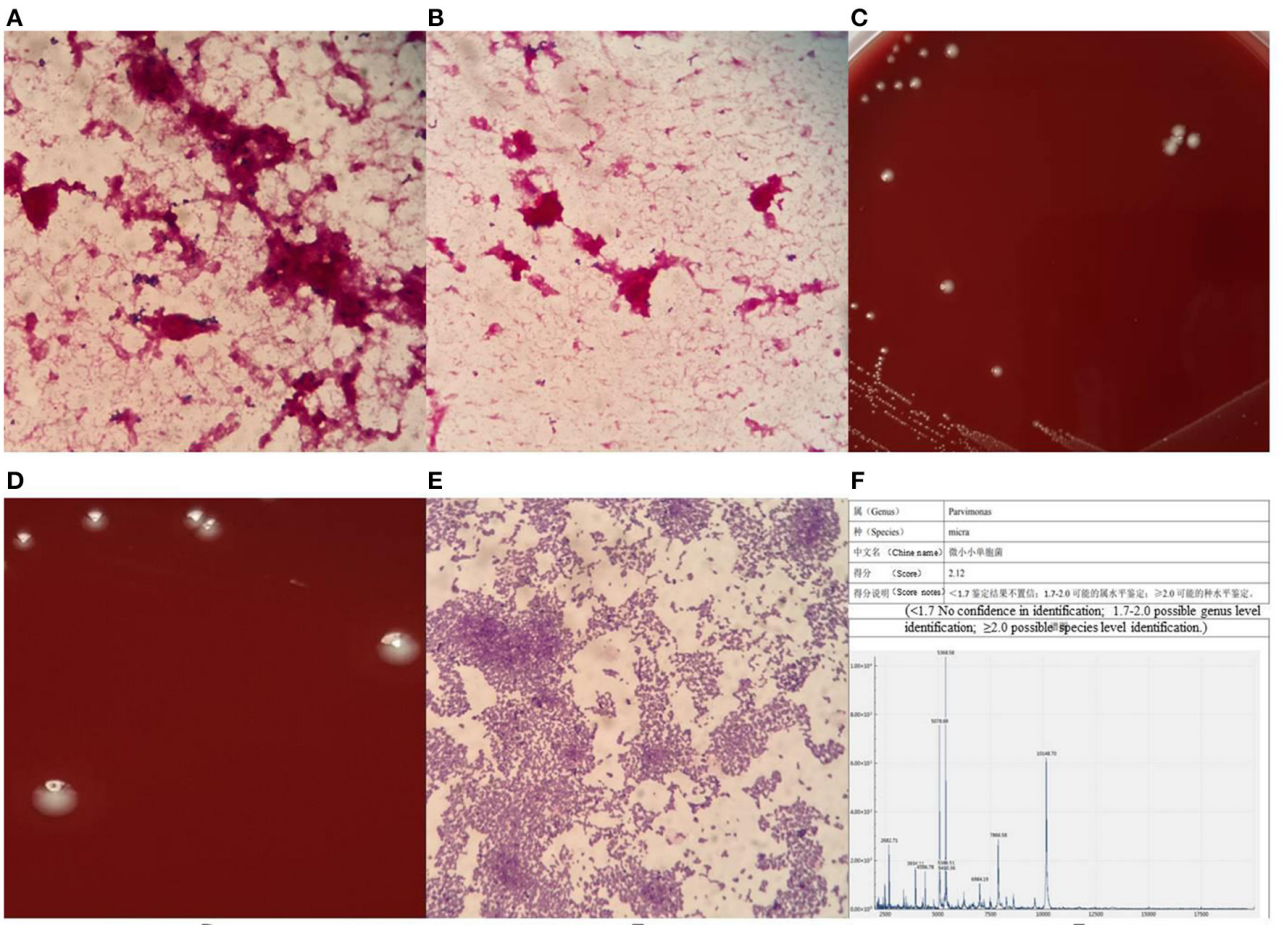
Gram stain of blood smear under microscope **(A)** × 1000, morphological distribution of *P. micra* **(B)** × 1000, morphological characteristics of colonies of *P. micra* **(C,D)**, Gram-stained morphology of *P. micra* colonies **(E)** × 1000, mass spectrometer identification result **(F)**.

Both sets of blood cultures from the patient were placed in a BACT/ALERT 3D automated blood culture instrument (BioMérieux, Inc., Durham, North Carolina, USA), and one anaerobic bottle was flagged as positive by the instrument after about 107 h, while the other one was flagged as positive after >120 h of culturing. We then streaked the blood from the anaerobic bottles onto a Columbia blood agar plate, MacConkey agar plate, and non-selective chocolate agar plate (all plates sourced from Zhengzhou Antu Biological Bioengineering Co., Zhengzhou, China) to plating, then placed the plates in a 35°C and 5% CO_2_ environment for culturing. Blood was then drawn again onto another Columbia blood agar plate to plating, and the plate was placed into an anaerobic environment for anaerobic culture. At the same time, a blood smear was prepared from anaerobic bottles for Gram staining microscopy. The microscopic investigation revealed that quite small Gram-positive cocci could be seen under the microscope (Figure 1A), distributed in groups or individually (Figure 1B). After 18 h of culturing, all plates in the aerobic environment were free of bacterial growth. However, tiny colonies were visible on the Columbia agar incubated in an anaerobic environment. This finding suggested that the patient had a bloodstream infection with anaerobic bacteria.

After 48 h of culturing, creamy white, round, moist colonies (there is only one type of colony) approximately 0.6 μm size were visible on the Columbia blood agar plate cultured in an anaerobic environment, with an areola-like formation around the colonies (Figures 1C,D), which was used as one of the identifying features of the bacterium. Pure colonies on the plate were then picked for Gram staining microscopy, and tiny Gram-positive cocci could be seen under the microscope, either in clusters or singly distributed (Figure 1E). Such colonies can easily be mis-stained as Gram-negative cocci, so the decolorization step of the Gram stain is vital, and the correct staining depends on the operator's experience. At this point, the strain was identified as *P. micra* using the Zhongyuan Huiji Micro Typer MALDI-TOF mass spectrometer (Chongqing Zhongyuan Huiji Biotechnology Co., Ltd., Chongqing, China) (Figure 1F). It took 1 week to finally identify the bacteria from the time the blood culture specimens were sent to the laboratory, which was too long a time period to report the pathogen to clinicians. In order to verify whether the strain was *P. micra*, we extracted DNA fragments from pure colonies and amplified 16S ribosomal RNA using the primers “27F” and “1492R.” Forward primer name 27F, 5'- AGT TTG ATC MTG GCT CAG−3', Reverse primer name 1492R, 5'- GGT TAC CTT GTT ACG ACT T−3', target fragment was about 1500 bp. Polymerase chain reaction (PCR) reaction system 50μL: PCR Mix 45μL, 2μL of each forward and reverse primer, 1μL of template DNA, dH_2_O (distilled water) 22 μL.The PCR reaction conditions: pre-denaturation at 98°C for 2 min; 35 cycles of 98°C for 10s, 56°C for 10s and 72°C for 10s/kb; extension at 72 °C for 5 min; After the reaction, 2μL of amplification products and 6μL of bromophenol blue were taken and the purity of PCR products were examined by 1% agarose gel electrophoresis. The contigExpress software was used to splice the sequences and remove the inaccurate ends, and then the spliced 16s rRNA sequences wereused for sequence comparison, which was performed by using the Basic Local Alignment Search Tool on the U.S. National Center for Biotechnology Information database website, and a fragment measuring 1,400 bp in length was selected for comparison and analyzed as *P. micra* (ATCC 33270; American Type Culture Collection, Manassas, VA, USA), with a similarity of 99.64% observed.

### Susceptibility to antibiotics

Because we had no resources for anaerobic bacteria *in vitro* antibiotics susceptibility testing at the time of this case, we had no way to obtain the antibiotics sensitivity results of this bacterium.

### Clinical treatment and prognosis

The patient was admitted to the intensive care unit on her first day in the hospital. After consultation with our orthopedic department, the patient was advised to rest in bed and symptomatically manage other symptoms as well as eliminate the cause of the infection, taking into account his current poor physical condition. The emergency chest computed tomography images indicated infection in both lungs, and piperacillin–tazobactam was given for the first 2 days as anti-infective treatment. However, the patient's infection did not improve at all. On the day after her admission to the hospital, her blood pressure suddenly started to drop, her blood pressure needed adrenaline to maintain. At this time, her blood pressure after using adrenaline was 104/62 mmHg; her heart rate reached 131 bpm; whole blood lactate reached 4.3 mmol/L; brain natriuretic peptide (BNP) reached 297 pg/mL, and her urine output was reduced. Combining these data with the above-mentioned information and the recorded high values of inflammatory indicators, the doctor suggested that the patient had infectious shock. Norepinephrine was continued giving to raise her blood pressure, and the doctor actively gave the patient fluid replacement and used furosemide for diuretic treatment. In the meantime, the antibiotic was changed to imipenem. On the fourth day of hospitalization, the patient developed hypoxemia, and she was intubated and treated with invasive mechanical ventilation. The patient's blood pressure was maintained at 122/62 mmHg by the administration of norepinephrine combined with M-hydroxylamine, and an assessment of inflammatory indicators revealed the following values: WBC count, 17.94 × 10^9^/L (percentage of neutrophils: 81.20%); procalcitonin concentration, 2.55 ng/mL; and CRP level, >200 mg/L. At this point the patient has very severe systemic inflammation. Unfortunately, the patient developed then generalized edema and on the fifth day of hospitalization and died of multiorgan failure due to septic shock. As the blood culture pathogenic results were not fed back to the clinician in a timely manner, coupled with the negative sputum culture results, the patient had to receive urgent empirical antibiotic treatment during hospitalization.

## Discussion

*P. micra* is a species of conditionally pathogenic anaerobic bacteria that can be found in various human mucous membranes. The size of *P. micra* is tiny (0.3–0.7 μm). *P. micra* was originally classified as *Peptostreptococcus micros*, then reclassified as *Micromonas micros* in 1999 and reclassified again as *P. micra* in 2006 (2). It is recognized as an oral pathogen (1); however, *P. micra* can also cause bloodstream infections, spinal infections, septic chest, and sepsis (3, 4). In most cases of infection with *P. micra*, the first symptom is usually lower back pain, and most patients are admitted to the hospital with back pain (4, 5). The patient in this case initially sought treatment at another hospital for lower back pain. At that time, she was probably already infected with *P. micra* in her lower back but had not yet developed any symptoms of pulmonary infection; as a result, she was likely viewed as a normal lumbar spine patient and treated using pain relief and traditional Chinese medicine physiotherapy. Even when the patient had obvious signs of lung infection and a bloodstream infection, several sputum culture results were still negative, and she did not immediately receive the correct antibiotic treatment. When the patient was admitted to our hospital, her lungs might have already been infected with *P. micra*. One study reported a 49-fold increase in tumor necrosis factor α release from macrophages following the binding of *P. micra* to endotoxin with *Haemophilus actinomycetemcomitans* (6); thus, it can be proved that *P. micra* has the ability to cause severe inflammation by stimulating macrophages to release various inflammatory factors. This may be the cause of systemic inflammation and sepsis outbreak in our patient.

In data from Tsuyoshi's study, the 30-day clinical mortality rate for bloodstream infections with *P. micra* was only about 3.7%, (4) but it is still worth drawing the attention of clinicians to this issue. The majority of patients infected with *P. micra* also have underlying medical conditions (2, 4, 7), which suggests that the prevalence of *P. micra* infection is linked to human immunity. In our case, the patient had a long history of rheumatoid arthritis and was unaware that she had diabetes. Both of these underlying conditions can cause severe disruption of and a reduction in the strength of a patient's autoimmune system, which likely led to infection of this patient's lower back with *P. micra* at first. For the treatment of *P. micra* infection, penicillin, metronidazole, and clindamycin are usually preferred (8). Our patient was initially treated with a 3-day infusion of piperacillin–tazobactam, but her infection was not controlled. The antibiotic of choice was later changed to meropenem, but the optimal timing for treatment might have passed by that point. Although both piperacillin–tazobactam and imipenem have some pharmacological effect on anaerobic bacteria, they were very ineffective in treating our patient's infection, and it is presumed that she may have also had a combination of other pathogenic infections. In a report by Yu et al., (9) a patient was treated with piperacillin–tazobactam and meropenem after lung infection with *P. micra*, but the initial outcome was unsatisfactory. The infection was finally treated with ornidazole after the pathogenic organism was identified by next-generation sequencing (NGS), and the lung infection was rapidly controlled. Thus, clinicians should consider the use of narrow-spectrum anti-anaerobic drugs in cases where conventional antibiotic therapy has failed.

In some reports of infections with *P. micra*, the final diagnosis is made by reference to the NGS results (9). NGS is the process of randomly fragmenting and splicing DNA, preparing sequencing libraries, and ultimately obtaining sequence information by performing extension reactions on the tens of thousands of clones in the library and detecting the corresponding signals (the core principle is sequencing while synthesizing). NGS can quickly provide results in as little as 24 h and is capable of detecting pathogens, including viruses, rickettsiae, and other pathogens, that cannot be detected by conventional culture methods. However, there were some shortcomings in our study and we regret that the doctors did not send the patients' blood specimens and respiratory specimens for NGS analysis in time to rule out mixed infections. Conventional anaerobic culture has a very low positive rate and takes a very long time, so new diagnostic techniques such as NGS should be considered when the source of infection is not clear and the infection is severe, as this approach can greatly reduce the time taken to find the causative agent, save time for the patient, and provide the clinician with the right treatment ideas.

## Data availability statement

The original contribution of this manuscript is available from (http://www.ncbi.nlm.nih.gov/bioproject/PRJNA859504).

## Author contributions

YJ wrote the manuscript. YZ reviewed the manuscript. YG, JL, and WQ collected the data for the manuscript. YJ and YZ share first authorship. All authors contributed to the article and approved the submitted version.

## Funding

This research was supported by the key technology innovation special of key industries of 246 Chongqing Science and Technology Bureau Fund (cstc2020jscx-fyzxX0021).

## Conflict of interest

The authors declare that the research was conducted in the absence of any commercial or financial relationships that could be construed as a potential conflict of interest.

## Publisher's note

All claims expressed in this article are solely those of the authors and do not necessarily represent those of their affiliated organizations, or those of the publisher, the editors and the reviewers. Any product that may be evaluated in this article, or claim that may be made by its manufacturer, is not guaranteed or endorsed by the publisher.
